# Community Awareness About Pediatric Cancer in Tanzania

**DOI:** 10.1200/GO.22.57000

**Published:** 2022-05-05

**Authors:** Erica Sanga, Doris Mbata, Elizabeth Msoka, Shabani Rajabu, Francis Karia, Hillary Sued, Kathryn Pollak, Kristin Schroeder

**Affiliations:** ^1^ National Institute of Medical Research—Lake Zone, Dar-es-Salaam, Tanzania; ^2^ National Institute of Medical Research Headquarters, Dar-es-Salaam, Tanzania; ^3^ Kilimanjaro Clinical Research Institute, Moshi, Tanzania; ^4^ Kilimanjaro Christian Medical University College, Moshi, Tanzania; ^5^ Kilimanjaro Christian Medical Center, Moshi, Tanzania; ^6^ Bugando Medical Centre, Mwanza, Tanzania; ^7^ Duke Clinical Research Institute, Durham, NC; ^8^ Duke University Medical Center, Durham, NC

## Abstract

Early hospital presentation and completion of treatment is crucial for better treatment outcomes in children with cancer. There are thousands of children who die of cancer each year without ever being diagnosed in Tanzania. To address this gap and inform childhood cancer awareness campaigns, it is important to understand the inherent cultural values and beliefs that influence health care seeking behavior among the Tanzanian community.

**METHODS:**

This was a descriptive qualitative study conducted in Mwanza, Kilimanjaro and Dar- es Salaam regions. Community members aged ≥ 18 years from three rural and three urban communities were purposively selected to participate in seven focus group discussions (each with 8-12 respondents) between March and June 2021. Data were transcribed, coded and analyzed by thematic content-analysis with the support of NVIVO software.

**RESULTS:**

A total of 72 participants were interviewed. Many had heard of breast or cervical cancer however, awareness of pediatric cancer was low. The belief that cancer in children is caused by witch craft led to initial evaluation by traditional healers leading to delayed presentation to the hospital. Additional community concerns included the cost of transportation and investigations, duration of treatment and community influence on families to abandon hospital treatment for spiritual or traditional treatment.

**CONCLUSION:**

Low community awareness about pediatric cancer, late hospital presentation and treatment abandonment remains a challenge in most parts of Tanzania. The common belief that childhood cancer is a result of witchcraft and superstition contributes to limited health seeking behavior, especially in the rural areas. Cultural and contextually relevant awareness interventions are needed to increase cancer knowledge and cancer health-seeking behavior in Tanzanian communities.

