# Erratum

**DOI:** 10.1200/GO.22.00184

**Published:** 2022-07-05

The meeting abstract by Sanga et al entitled “Community Awareness About Pediatric Cancer in Tanzania” (*JCO Glob Oncol* 10.1200/GO.22.57000) was published online May 5, 2022, with errors.

In the affiliation list, the following should have been included:

- ^**4**^**Kilimanjaro Christian Medical University College, Moshi, Tanzania**
- ^**5**^**Kilimanjaro Christian Medical Center, Moshi, Tanzania**

In the author list, `Elizabeth Msoka, MSc^3^ should have been Elizabeth Msoka, MSc^3,4,5^. The remaining author affiliations should have been Shabani Rajabu, MA^6^; Francis Karia, MBA, MSc-PH^7^; Hillary Sued, BS^6^; Kathryn Pollak, PhD^8^; and Kristin Schroeder, MD, MPH^8^ and the full affiliation list updated to:

^1^National Institute of Medical Research—Lake Zone, Dar-es-Salaam, Tanzania

^2^National Institute of Medical Research Headquarters, Dar-es-Salaam, Tanzania

^3^Kilimanjaro Clinical Research Institute, Moshi, Tanzania

^4^Kilimanjaro Christian Medical University College, Moshi, Tanzania

^5^Kilimanjaro Christian Medical Center, Moshi, Tanzania

^6^Bugando Medical Center, Mwanza, Tanzania

^7^Duke Clinical Research Institute, Durham, NC

^8^Duke University Medical Center, Durham, NC

This has been corrected as of July 5, 2022. The authors apologize for the errors.

